# Spectrally resolved autofluorescence imaging in posterior uveitis

**DOI:** 10.1038/s41598-022-18048-4

**Published:** 2022-08-29

**Authors:** Maximilian W. M. Wintergerst, Nicholas R. Merten, Moritz Berger, Chantal Dysli, Jan H. Terheyden, Enea Poletti, Frank G. Holz, Valentin S. Schäfer, Matthias Schmid, Thomas Ach, Robert P. Finger

**Affiliations:** 1 Department of Ophthalmology, University Hospital Bonn, Venusberg-Campus 1, 53127 Bonn, Germany; 2 Department of Medical Biometry, Informatics and Epidemiology, University of Bonn/University Hospital Bonn, Bonn, Germany; 3 Department of Ophthalmology, Inselspital, Bern University Hospital, University of Bern, Bern, Switzerland; 4 CenterVue, Padua, Italy; 5 Clinic of Internal Medicine III, Oncology, Hematology, Rheumatology, and Clinical Immunology, University Hospital Bonn, Bonn, Germany

**Keywords:** Diagnostic markers, Retina, Imaging and sensing, Acute inflammation, Chronic inflammation, Imaging, Fluorescence imaging, Optical imaging, Eye diseases, Retinal diseases, Uveal diseases

## Abstract

Clinical discrimination of posterior uveitis entities remains a challenge. This exploratory, cross-sectional study investigated the green (GEFC) and red emission fluorescent components (REFC) of retinal and choroidal lesions in posterior uveitis to facilitate discrimination of the different entities. Eyes were imaged by color fundus photography, spectrally resolved fundus autofluorescence (Color-FAF) and optical coherence tomography. Retinal/choroidal lesions’ intensities of GEFC (500–560 nm) and REFC (560–700 nm) were determined, and intensity-normalized Color-FAF images were compared for birdshot chorioretinopathy, ocular sarcoidosis, acute posterior multifocal placoid pigment epitheliopathy (APMPPE), and punctate inner choroidopathy (PIC). Multivariable regression analyses were performed to reveal possible confounders. 76 eyes of 45 patients were included with a total of 845 lesions. Mean GEFC/REFC ratios were 0.82 ± 0.10, 0.92 ± 0.11, 0.86 ± 0.10, and 1.09 ± 0.19 for birdshot chorioretinopathy, sarcoidosis, APMPPE, and PIC lesions, respectively, and were significantly different in repeated measures ANOVA (*p* < 0.0001). Non-pigmented retinal/choroidal lesions, macular neovascularizations, and fundus areas of choroidal thinning featured predominantly GEFC, and pigmented retinal lesions predominantly REFC. Color-FAF imaging revealed involvement of both, short- and long-wavelength emission fluorophores in posterior uveitis. The GEFC/REFC ratio of retinal and choroidal lesions was significantly different between distinct subgroups. Hence, this novel imaging biomarker could aid diagnosis and differentiation of posterior uveitis entities.

## Introduction

Posterior uveitis is considered an orphan disease and encompasses a heterogeneous group of diseases, many of which are not completely understood, and their clinical discrimination and management remains a challenge^1^. Therefore, it is crucial to explore novel diagnostic methods to increase our understanding and facilitate discrimination of the different entities. Fundus autofluorescence (FAF) is a non-invasive imaging technique that is widely used for detection and monitoring of retinal diseases^2–5^. FAF uses the fluorescence properties of intrinsic fluorophores in order to assess metabolism and vitality of the retinal pigment epithelium (RPE) and photoreceptor layers. Previous studies have shown that inflammatory activity can be associated with the appearance of retinal and choroidal lesions on FAF: in several uveitis entities, active retinal lesions are hyperautofluorescent, whereas inactive lesions are hypoautofluorescent^6,7^.

A novel confocal 450 nm excitation wavelength blue-light FAF device has been introduced recently, which, in contrast to the established FAF modalities, allows for spectrally resolved FAF (Color-FAF) detection and stratification of green (GEFC, 500–560 nm) and red emission fluorescent components (REFC, 560–700 nm). The benefit of this novel FAF technique in terms of novel insights into pathogenesis and providing a marker for disease progression has already been demonstrated in other retinal diseases^8–12^ and it might also allow additional insights into retinal and choroidal lesions in posterior uveitis.

The aim of this exploratory study was to evaluate Color-FAF imaging as diagnostic imaging biomarker in different posterior uveitis entities.

## Methods

### Participants

This retrospective study included patients from uveitis and other outpatient clinics at the University Hospital Bonn, Department of Ophthalmology, Germany, with posterior uveitis in agreement with the diagnostic criteria of the SUN working group^13,14^. It enrolled acute posterior multifocal placoid pigment epitheliopathy (APMPPE), ocular sarcoidosis mimicking birdshot chorioretinopathy in terms of non-pigmented bright lesions, punctate inner choroidopathy (PIC), birdshot chorioretinopathy, toxoplasmosis chorioretinits, multifocal choroiditis and panuveitis (MCP), presumed ocular histoplasmosis syndrome (POHS), or serpiginous choroiditis. Patients were included from October 2017 until March 2021. The study was conducted in adherence to the Declaration of Helsinki. Ethical approval was obtained from the ethics committee of the University of Bonn (approval ID 548/20). Informed consent is waived by the ethics committee of the University of Bonn. Exclusion criteria were idiopathic forms of posterior uveitis, reduced image quality (blurred image or shadowing), significant vitreous opacities, advanced cataract, inability to perform the examination, or any other retinal disease except for the stigmata of posterior uveitis. Subgroups of posterior uveitis entities with less than 5 eyes were not included in this study.

### Image acquisition

All patients underwent pupil dilatation (using 0.5% tropicamide, 2.5% phenylephrine), were examined by an ophthalmologist and imaged by color-fundus photography (CFP, 440–650 nm), FAF with blue excitation light (440–475 nm with a peak at 450 nm) and infrared reflectance (825–870 nm, 60° × 55°, image resolution 3680 × 3288 pixel, Eidon TrueColor Confocal Scanner, CenterVue, Padua, Italy), and enhanced depth spectral-domain optical coherence tomography (SD-OCT, 30° and 55°, at least 40 automatic real-time tracking repetitions, at least 60 B scans, image resolution 496 pixels per A-Scans and 768 A-Scans per B-Scan, Spectralis HRA + OCT, Heidelberg Engineering, Heidelberg, Germany). Clinical data were obtained from the medical charts. Eidon FAF images were exported as grayscale and Color-FAF images (detection range 500–750 nm). Color-FAF images were generated by the use of a barrier filter with a cut-off at 500 nm and by the device by separating GEFC and REFC. If multiple images were available, images with the best image quality were selected for analyses.

### Data analyses

**Figure 1:**
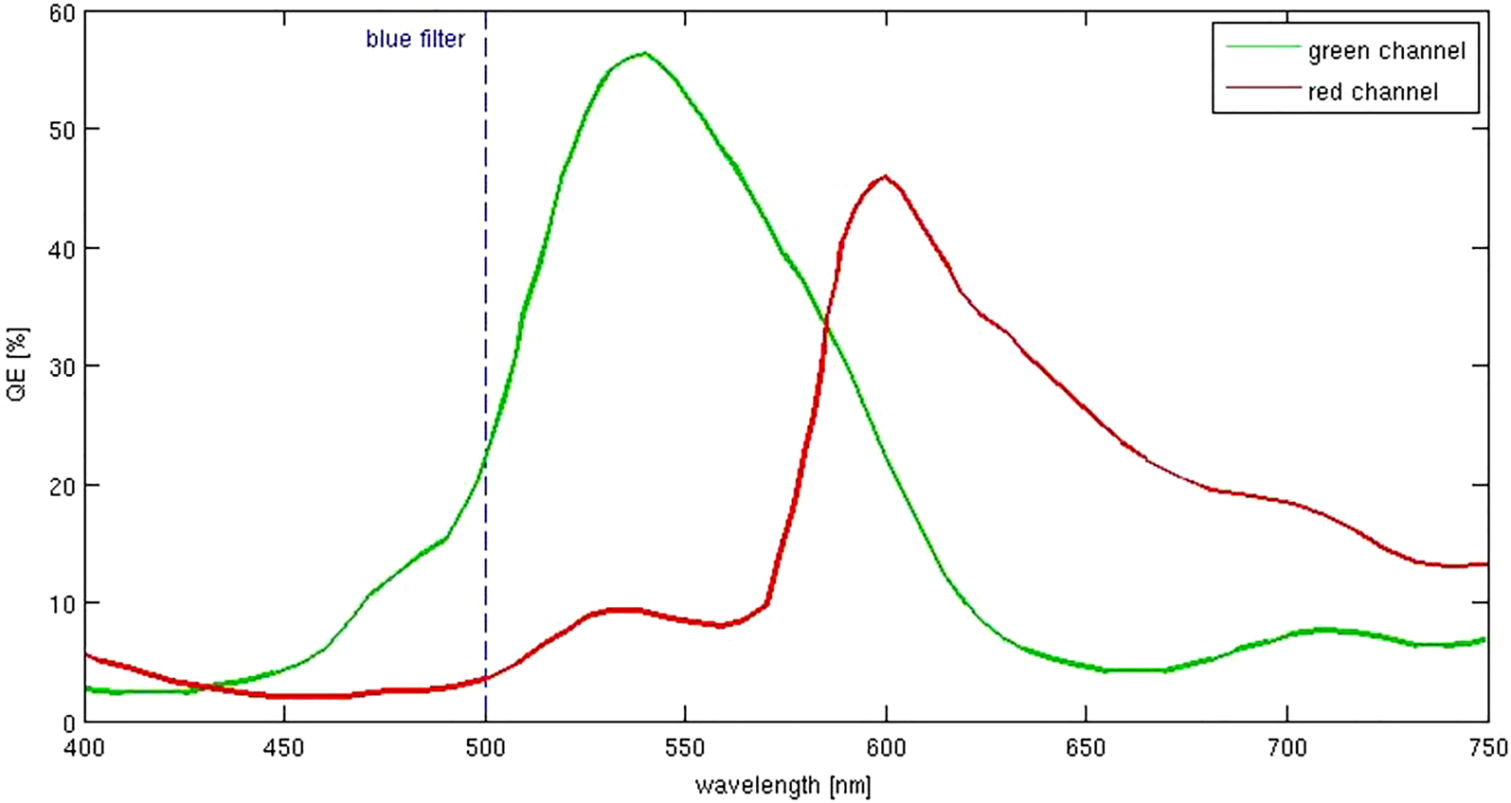
Eidon Autofluorescence sensor quantum efficiency. Each wavelength from 500 to 750 nm contributes to both, green and red channels with different efficiencies. The single-color component (red or green) of a pixel has to be interpreted as the total contribution of all wavelengths collected by the sensor after they are multiplied by their respective quantum efficiency (QE). The dashed line illustrates the barrier filter with the cut-off at 500 nm.

Inflammatory activity was defined on an eye-level in birdshot chorioretinopathy and ocular sarcoidosis and on a lesion-level in APMPPE, PIC, Toxoplasmosis chorioretinitis, MCP, POHS, and serpiginous choroiditis (inflammatory activity grading available in Supplementary Table S1)^15–23^. In general, the synopsis of all clinical findings was determinant for the definition of inflammatory activity. Both, inflammatory active and inactive eyes were included in this exploratory study. Image processing for quantitative analysis was performed with ImageJ (National Institutes of Health, Bethesda, Maryland; available at (“https://imagej.nih.gov/ij/”)^24^ using the “Split Channels” function for separation of GEFC and REFC channels. The autofluorescence intensity of every retinal and choroidal lesion in the Color-FAF image was manually measured separately for GEFC and REFC channel using ImageJ and the Color-FAF characteristics of each lesion were determined by calculating the GEFC/REFC ratio for all entities with at least 100 lesions. Subgroups with less than 100 lesions were only included in the qualitative analyses. Color-FAF images were imported into the analysis software “FAF Color Segmentation Tool” (CenterVue, Padova, Italy) for analysis of the predominant wavelength component of the Color-FAF, as described in previous studies^9,10^. Using this software, an intensity-normalized Color-FAF image was generated for illustration of differences in FAF emission wavelength. The color of each pixel of the intensity-normalized Color-FAF image was derived from the main emitted wavelength of the pixel by the following technique: each pixel of the original image data had a specific intensity in the REFC and the GEFC channel. The intensity of a pixel in the REFC and GEFC channel was dependent on the contribution of emitted wavelengths by the pixel and their quantum efficiency (Fig. 1). The resulting color of the pixel was calculated from the relationship between the distribution of intensities in REFC and GEFC. Using a fitted exponential function based on empirical data of fluorophores with known emission spectra, the intensity-normalized Color-FAF image was generated.

### Statistical analyses

Statistical analyses were performed using R (R: A Language and Environment for Statistical Computing, version 3.4.1, R Core Team, R Foundation for Statistical Computing, Vienna, Austria, 2016). Repeated measures ANOVA was used for the comparison of the ratios of GEFC/REFC in different posterior uveitis entities. Furthermore, multivariable regression analysis, using mixed models to account for the clustered structure of the data (large number of lesions and repeated measures within one eye), was performed to determine the influence of lens status and inflammatory activity on the GEFC/REFC ratio.

## Results

### Demographics and clinical characteristics

A total of 76 eyes of 45 patients was included in the study. Four eyes of 3 patients were excluded due to reduced Color-FAF image quality and 4 eyes of 2 patients because of coincident age-related macular degeneration. Characteristics of the patients and uveitis entities are provided in Table 1. There were no relevant lens opacities present in any of the included phakic eyes. Three out of 13 eyes with ocular sarcoidosis (22 lesions), 2 out of 9 eyes with birdshot chorioretinopathy (23 lesions), 2 out of 22 eyes with APMPPE (29 lesions), 1 out of 12 eyes with PIC (11 lesions), 2 out of 8 eyes with toxoplasmosis (2 lesions), and 4 out of 5 eyes with serpiginous choroiditis (10 lesions) showed clinically active inflammation at the time of imaging. In the included eyes with MCP and POHS there were no active lesions present.

**Table 1:** Characteristics of the sample.

	Mean ± SD (Range) or N (%)
Age (years)	48 ± 17 (17–81)
Sex (male)	36 (46%)
Lens status (phakic)	61 (78%)
VA (logMAR)	0.26 ± 0.41 (0.0–1.7)
Duration of uveitis (months)	72 ± 86 (0–329)
**Posterior uveitis entities (eyes (lesions))**	
APMPPE	22 (338)
Ocular sarcoidosis	13 (124)
PIC	12 (129)
Birdshot chorioretinopathy	9 (151)
Toxoplasmosis chorioretinitis	8 (14)
MCP & POHS	7 (74)
Serpiginous choroiditis	5 (15)

*SD* Standard deviation, *VA* (best-corrected) Visual acuity, *PIC* Punctate inner choroidopathy, *APMPPE* Acute posterior multifocal placoid pigment epitheliopathy, *MCP* Multifocal choroiditis and panuveitis, *POHS* Presumed ocular histoplasmosis syndrome.

### Qualitative analysis of color- FAF images and association with OCT

**Figure 2:**
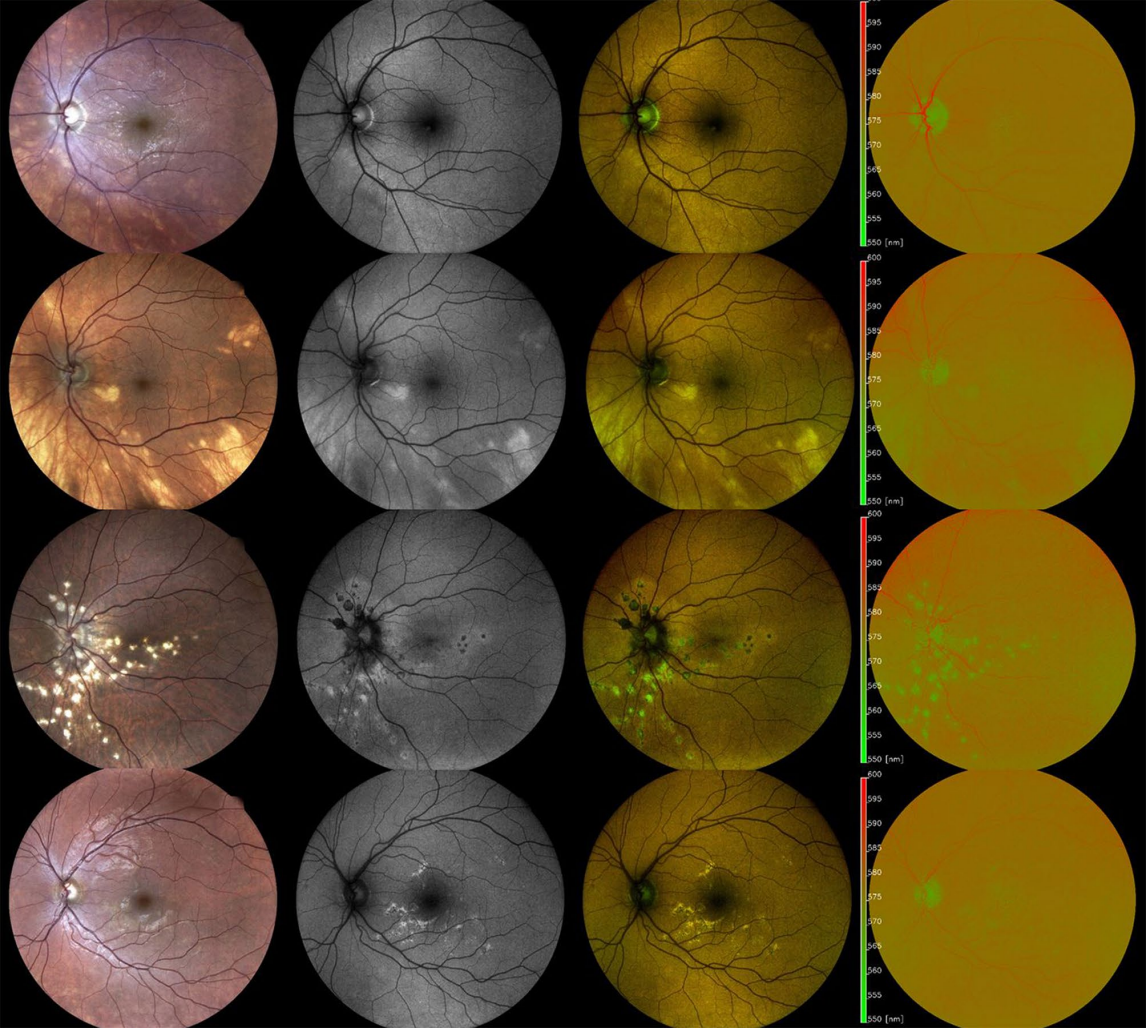
Comparison of birdshot chorioretinopathy, ocular sarcoidosis, punctate inner choroidopathy (PIC), and acute posterior multifocal placoid pigment epitheliopathy (APMPPE) on spectrally resolved autofluorescence (Color-FAF) imaging. An exemplary eye with birdshot chorioretinopathy (first row), ocular sarcoidosis (second row), PIC (third row), and APMPPE (fourth row) are illustrated. From left to right: color fundus photography (CFP), grayscale fundus autofluorescence image (grayscale FAF), spectrally resolved fundus autofluorescence (Color-FAF) image, intensity-normalized Color-FAF. Lesions of eyes affected by ocular sarcoidosis were more apparent than in eyes with birdshot chorioretinopathy on grayscale FAF, Color-FAF, and intensity-normalized Color-FAF. Lesions of eyes with PIC were clearly apparent in all four modalities. Lesions of eyes affected by APMPPE were hardly visible on CFP and on intensity-normalized Color-FAF, whereas they were easily definable on Color-FAF and grayscale FAF.

The typical discrete lesions on CFP in birdshot chorioretinopathy were hardly or not at all determinable on Color-FAF, grayscale FAF and intensity-normalized Color-FAF. Lesions typical of ocular sarcoidosis however, appeared hyperautofluorescent on grayscale FAF and showed a predominantly green emission wavelength of low visibility on the intensity-normalized Color-FAF (Fig. 2). OCT images revealed choroidal thinning in fundus areas corresponding to the white dots in all 13 eyes with ocular sarcoidosis both in active and inactive disease. In contrast, there was no notable choroidal thinning in fundus areas corresponding to birdshot chorioretinopathy lesions both in active and inactive disease, except for one eye (Supplementary Fig. S1). Lesions of APMPPE, typically barely visible on CFP, appeared predominantly greenish on intensity-normalized Color-FAF. On grayscale FAF and Color-FAF, lesions were clearly identifiable. APMPPE lesions showed a disrupted ellipsoid and interdigitation zone with a preserved RPE and Bruch’s membrane on OCT (in 19 of 22 eyes) and RPE atrophy (in 3 of 22 eyes). PIC lesions were well demarcated in all four imaging modalities. In intensity-normalized Color-FAF, PIC lesions appeared strongly greenish (Fig. 2). An interruption of outer retinal layers, including RPE and Bruch’s membrane, was evident on OCT scans corresponding to fundus areas affected by PIC in all 12 eyes with PIC (Supplementary Fig. S2).

**Figure 3:**
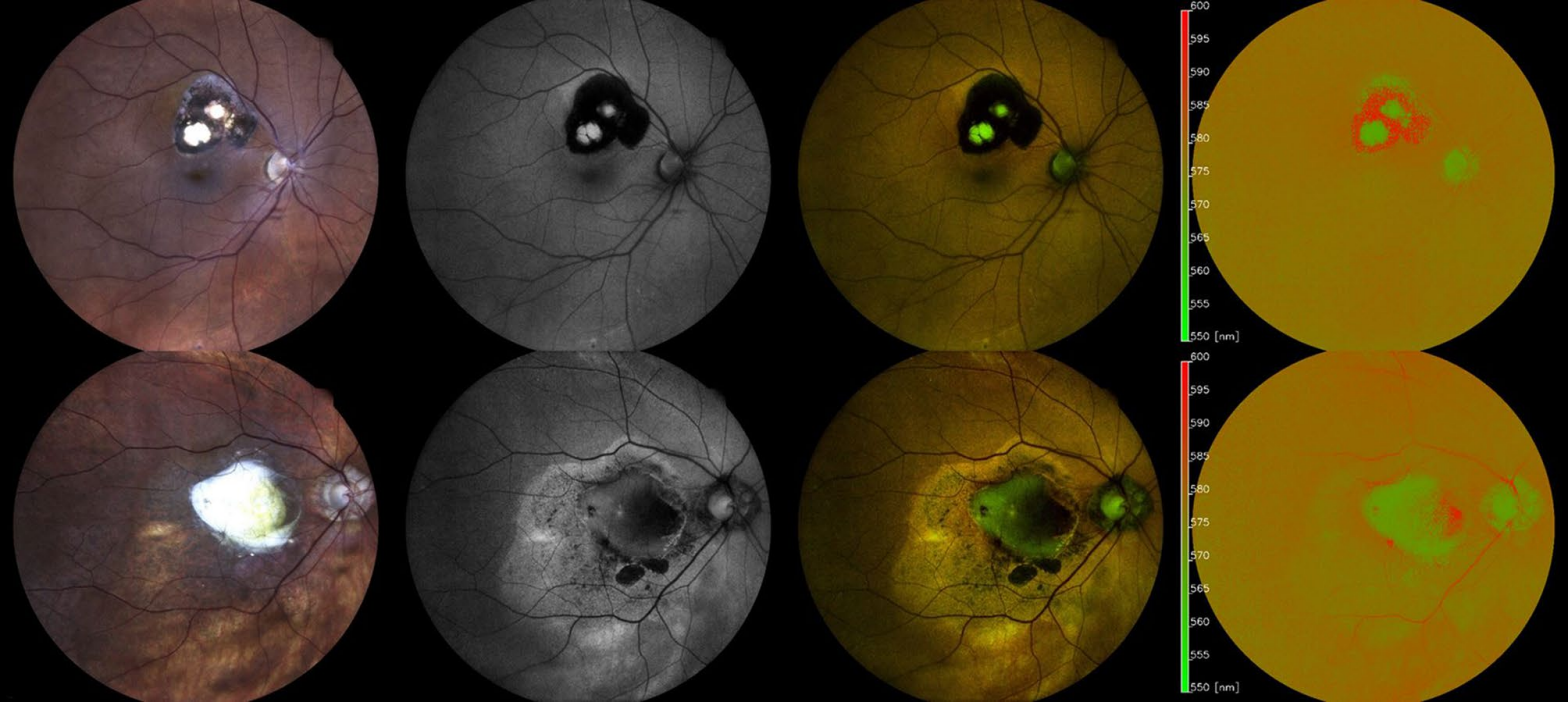
Sources of green and red emission fluorescent components in posterior uveitis. Exemplary eyes with a pigmented scar in toxoplasmosis chorioretinitis (top row) and with macular neovascularization (MNV) in multifocal choroiditis and panuveitis (MCP; bottom row). From left to right: color fundus photography (CFP), grayscale fundus autofluorescence (grayscale FAF), spectrally resolved fundus autofluorescence (Color-FAF) image, intensity-normalized Color-FAF. Pigmented parts of the toxoplasmosis lesion features predominantly red emission fluorescent components (REFC) on intensity-normalized Color-FAF. On grayscale FAF and Color-FAF the pigmented parts were hypoautofluorescent and indistinguishable from parts of the lesion without pigmentation. The MNV in the eye with MCP featured mainly GEFC on Color-FAF and intensity-normalized Color-FAF.

Qualitative image analysis showed that optic nerve head and RPE atrophy featured predominantly GEFC and retinal vessels predominantly REFC. Pigmented retinal lesions, present in 20 eyes (14 patients), featured mainly REFC on intensity-normalized Color-FAF, whereas macular neovascularizations (MNVs), present in 7 eyes (5 patients), featured mainly GEFC (Fig. 3).

### Quantitative analysis of color-FAF images

**Figure 4:**
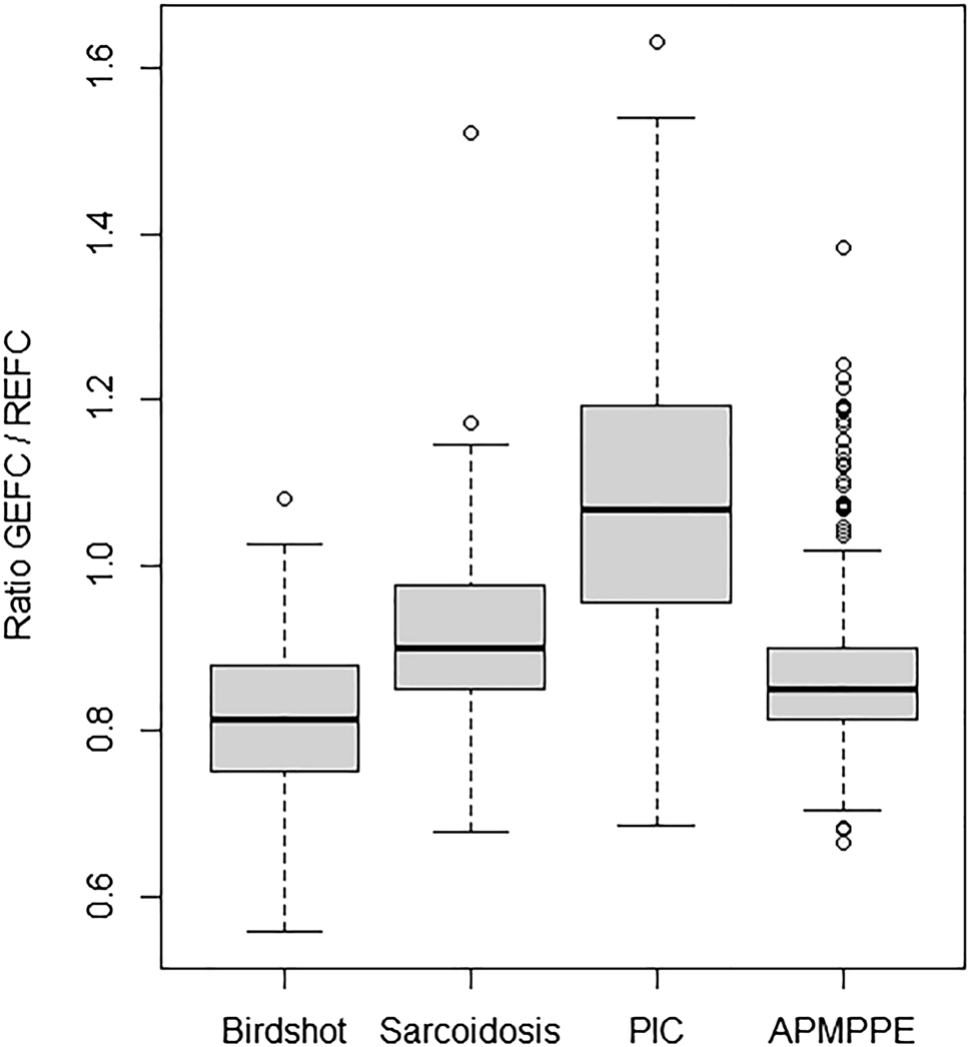
Quantitative analysis of GEFC/REFC ratio for posterior uveitis entities. Birdshot chorioretinopathy (left), ocular sarcoidosis (middle-left), punctate inner choroidopathy (PIC; middle-right), acute posterior multifocal placoid pigment epitheliopathy (APMPPE; right). Values over 1.5 interquartile range below the first quartile or above the third quartile were defined as outliers. Multivariable regression analysis showed significant differences (*p* < .05) among all posterior uveitis entities except for the comparison of birdshot chorioretinopathy and APMPPE (*p* = .07).

The ratio of autofluorescence intensity in the GEFC and REFC channel was calculated for lesions in eyes with confirmed diagnosis of posterior uveitis of subgroups with at least 100 lesions: ocular sarcoidosis, birdshot chorioretinopathy, APMPPE, and PIC. In total 742 lesions were analyzed: 124 lesions from eyes with sarcoidosis, 151 lesions from eyes with birdshot chorioretinopathy, 129 lesions from eyes with PIC, and 338 lesions from eyes with APMPPE. In ocular sarcoidosis the mean GEFC/REFC ratio was 0.92 (± 0.11), for birdshot chorioretinopathy the mean ratio was 0.82 (± 0.10), for APMPPE the mean ratio was 0.86 (± 0.10), and for PIC the mean ratio was 1.09 (± 0.19) (Fig. 4). Repeated measures ANOVA showed a significant difference for the comparison of these 4 posterior uveitis entities (*p* < 0.0001). Multivariable regression analysis using mixed models (and adjusting for lens status and inflammatory activity) showed significant differences for all posterior uveitis entities except for the comparison of birdshot chorioretinopathy and APMPPE (*p* = 0.07). Furthermore, lens status and inflammatory activity had no significant influence on the GEFC/REFC ratio (*p* = 0.98 and *p* = 0.33) in each subgroup (Supplementary Table S2).

Qualitative and quantitative findings on Color-FAF, intensity-normalized Color-FAF and OCT are summarized in Supplementary Table S3.

## Discussion

To the best of our knowledge, this is the first study evaluating spectrally resolved fundus autofluorescence in patients with uveitis. We demonstrate involvement of both short- and long-wavelength emitting fluorophores in retinal and choroidal lesions in posterior uveitis, significantly different GEFC/REFC ratios in lesions between different posterior uveitis entities, and postulate previously unknown sources of GEFC and REFC. Spectrally resolved fundus autofluorescence introduces novel imaging biomarkers and may aid diagnosis of posterior uveitis entities.

The use of conventional FAF imaging has already been evaluated for various retinal diseases^5,25–27^ and previous studies have shown that conventional FAF imaging can aid monitoring in posterior uveitis. FAF allows for identification of more chorioretinal lesions than conventional color fundus photography, it can reveal a greater extent of RPE damage, and it facilitates the differentiation between active and inactive parts of inflammatory lesions^2,7,28,29^. The autofluorescence detected in short-wavelength FAF emission (excitation: 488 nm) has its assumed origin mainly in bisretinoids of RPE lipofuscin, whereas long-wavelength FAF emissions (excitation: 785 nm) have their assumed origin mainly in melanin of the RPE and choroid^30^. Color-FAF imaging uses an excitation wavelength of 450 nm and is thought to excite more intensely minor fluorophores besides lipofuscin^8,31^. Therefore, it offers additional information and might provide novel insight into the pathophysiology of different posterior uveitis entities.

Despite multimodal imaging and a variety of available laboratory analyses, the diagnosis of posterior uveitis can remain challenging. Little is known about the pathophysiology of distinct posterior uveitis entities, and their heterogeneous manifestations can make it difficult to come to an accurate diagnosis^32^. For example, birdshot chorioretinopathy and ocular sarcoidosis can look alike, as both present with granulomatous inflammation of the choroid and with retinal vasculitis^33,34^. Our study shows that Color-FAF imaging may aid in differentiating between birdshot chorioretinopathy and ocular sarcoidosis based on significantly different GEFC/REFC ratios and by means of the intensity-normalized Color-FAF image.

A possible explanation for the observed differences in autofluorescence intensity and different main emission wavelengths might be the association with choroidal thinning, as we observed a thinner choroid on OCT in areas with lesions in ocular sarcoidosis in contrast to birdshot chorioretinopathy and to not affected fundus areas. This finding suggests that the sources of shortwave autofluorescence (GEFC) may be located in deeper choroidal layers or scleral tissue. The sources of GEFC and REFC have been discussed in previous studies: structures known to feature predominantly GEFC are fundus areas without underlying RPE (e.g. retinal scars, RPE atrophies, optic nerve head) while retinal vessels have been shown as a main source for REFC^8,9^. We were able to identify additional sources of REFC and GEFC: pigmented lesions appeared reddish on the intensity-normalized Color-FAF and MNVs greenish. Minor fluorophores, such as advanced glycation end products (AGE), flavin adenine dinucleotide and collagen/elastin are the main intrinsic short-wavelength fluorophores that are thought to cause GEFC autofluorescence^8,31^. Collagen and elastin are known to be principal parts of subchoroidal tissue, tenon’s capsule, and sclera^35,36^, hence, may be important intrinsic fluorophores contributing to GEFC signal in presence of choroidal thinning in retinal and choroidal lesions due to posterior uveitis.

This hypothesis is further supported by our findings in PIC. PIC lesions showed a predominantly green emission wavelength on intensity-normalized Color-FAF and corresponding OCT scans revealed a disruption of RPE and Bruch’s membrane, congruently with previous literature^37,38^. In contrast, APMPPE lesions appeared rather equally greenish and reddish, and less well defined on intensity-normalized Color-FAF, and showed preserved RPE and Bruch’s membrane on OCT^39^. It is possible that RPE disruption and concomitant reduction of RPE lipofuscin in affected fundus areas leads to stronger excitation of scleral/subchoroidal fluorophores and subsequently more intense emission of GEFC.

AGEs are considered as important source of GEFC in age-related macular degeneration^8^. There is a reasonable suspicion that AGEs play an important role in pathogenesis of chronic posterior uveitis as well, since patients with endogenous posterior uveitis entities show elevated serum levels of AGEs. Accumulation of AGEs in human retina is assumed based on a mouse model confirming elevated levels of retinal AGEs in mice due to induced human endogenous uveitis^40^. Thus, it is reasonable to assume that AGEs may contribute decisively to predominant short-wavelength emissions in lesions caused by endogenous posterior uveitis. Yet, further histopathological investigations are required to determine the involvement of AGE in posterior uveitis as well as to confirm our hypotheses.

The limitations of our study are a relatively small sample size and the exploratory, cross-sectional design. Strengths of our study include the introduction of the novel parameter GEFC/REFC ratio to characterize retinal and choroidal lesions by Color-FAF. Color-FAF imaging using the described device is an automated process and therefore easily reproducible and practicable in a clinical setting^10^. Owing to strict inclusion criteria we did not include any eyes with relevant media opacities, hence, minimized the influence of this potential confounder on our results. It is important to note that our quantifications aimed to analyze mean GEFC/REFC ratio for complete lesions, and therefore did not assess differences in emission spectra within single lesions. Future studies should analyze differences in GEFC/REFC ratio within single lesions, as different uveitis entities and active and inactive lesions might have distinct intra-lesion GEFC/REFC ratio distributions. Furthermore, a method allowing for proper quantification of Color-FAF intensity similar to quantitative autofluorescence imaging using an internal reference would be useful for future investigations^12^.

Our study introduced GEFC/REFC ratios as a novel imaging biomarker for posterior uveitis using spectrally resolved fundus autofluorescence and provides means for differentiation of posterior uveitis entities, which can be difficult to distinguish otherwise. Furthermore, we found previously unknown sources of REFC and GEFC. Longitudinal studies are warranted to evaluate the use of spectrally resolved FAF for diagnosis and monitoring of posterior uveitis.

## Supplementary Information

Supplementary Information.

## Data Availability

The datasets generated and analyzed during the current study are not publicly available due to data protection regulations but are available from the corresponding author on reasonable request.
