# Supplementary material for: Spectrally resolved autofluorescence imaging in posterior uveitis
Source: Sci Rep. 2022 Aug 29;12:14337. doi: 10.1038/s41598-022-18048-4 (PMC9424200; doi:10.1038/s41598-022-18048-4)
Supplement: Supplementary file 1 — Supplementary Information. [file 41598_2022_18048_MOESM1_ESM.pdf]

Spectrally Resolved Autofluorescence Imaging in Posterior Uveitis

Maximilian W. M. Wintergerst*,[1], Nicholas R. Merten*,[1], Moritz Berger[2], Chantal Dysli[3], Jan H. Terheyden[1], Enea Poletti[4], Frank G. Holz[1], Valentin S. Schäfer[5], Matthias Schmid[2], Thomas Ach[1], Robert P. Finger[1]

*** contributed equally**

[1] Department of Ophthalmology, University of Bonn, Bonn, Germany

[2] Department of Medical Biometry, Informatics and Epidemiology, University of Bonn / University Hospital Bonn, Bonn, Germany

[3] Department of Ophthalmology, Inselspital, Bern University Hospital, University of Bern, Bern, Switzerland

[4] CenterVue, Padua, Italy

[5] Clinic of Internal Medicine III, Oncology, Hematology, Rheumatology, and Clinical Immunology, University Hospital Bonn, Bonn, Germany

Supplementary Table S1. Inflammatory activity grading

	active	inactive
Ocular sarcoidosis & Birdshot chorioretinopathy	presence of macular edema<br>vitreous cells ≥ 2+<br>active vasculitis<br>new lesions	no macular edema<br>vitreous cells < 2+<br>no signs of active vasculitis
APMPPE	fuzzy borders, creamy, yellowish / greyish color on CFP<br>hyperautofluorescence on FAF<br>subretinal fluid on OCT	sharply defined borders on CFP<br>pigment granularity on CFP<br>hypoautofluorescence on FAF<br>no subretinal fluid on OCT<br>RPE hyperreflectivity on OCT
PIC	fuzzy borders, creamy, yellowish / greyish color on CFP<br>hyperautofluorescent retina surrounding<br>hypoautofluorescent lesion on FAF	sharply defined borders, punched-out appearance on CFP<br>pigmentation on CFP<br>hypoautofluorescence on FAF
Toxoplasmosis chorioretinitis &<br>MCP & POHS &<br>Serpiginous choroiditis	fuzzy borders, creamy, yellowish / greyish color on CFP<br>hyperautofluorescence on FAF	sharply defined borders on CFP<br>pigmentation on CFP<br>hypoautofluorescence on FAF

APMPPE = acute posterior multifocal placoid pigment epitheliopathy; PIC = punctate inner choroidopathy; MCP multifocal choroiditis and panuveitis; POHS = presumed ocular histoplasmosis syndrome; CFP = color fundus photography; OCT = optical coherence tomography; FAF = fundus autofluorescence; Ocular sarcoidosis and birdshot chorioretinopathy were graded on an eye-level, whereas APMPPE, PIC, toxoplasmosis chorioretinitis, MCP, POHS, and serpiginous choroiditis were graded on a lesion level;
Sources: Birdshot & Sarcoidosis:[1-3], APMPPE: [4,5]
PIC: [6,7], Toxoplasmosis, APMPPE, MCP: [8-10]

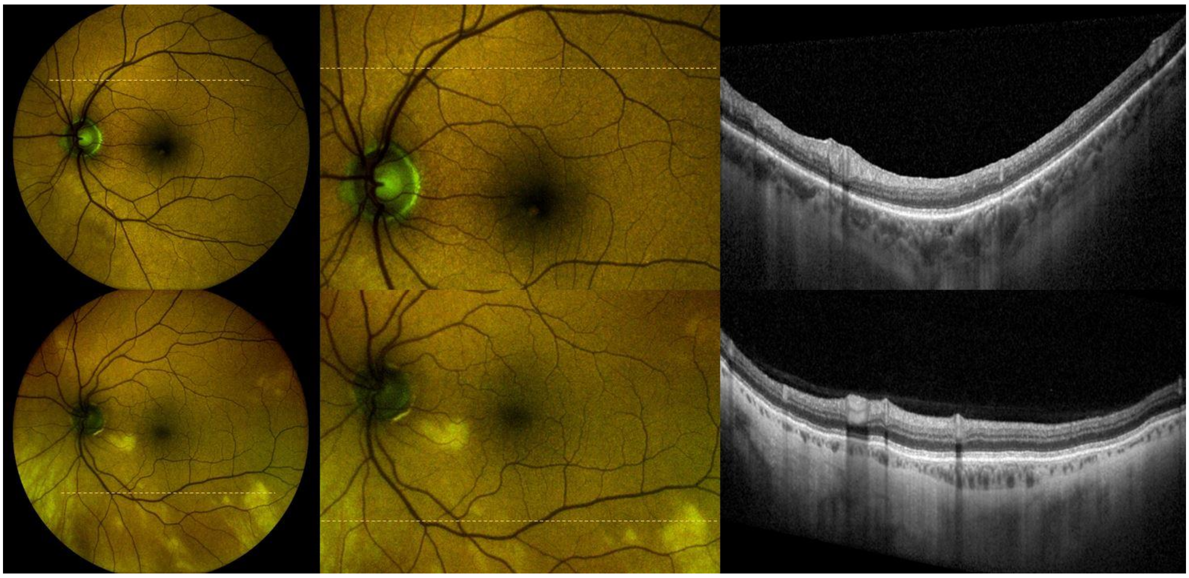

Supplementary Figure S1. Exemplary comparison of OCT and Color-FAF for one eye with birdshot chorioretinopathy and ocular sarcoidosis

One exemplary eye with birdshot chorioretinopathy (top row) and ocular sarcoidosis (bottom row) are shown on Color-FAF and on OCT. Lesions due to birdshot chorioretinopathy are hardly visible on Color-FAF and show no significant choroidal thinning on OCT. Lesions secondary to ocular sarcoidosis are easily identifiable on Color-FAF and show general and lesion-specific choroidal thinning on OCT.

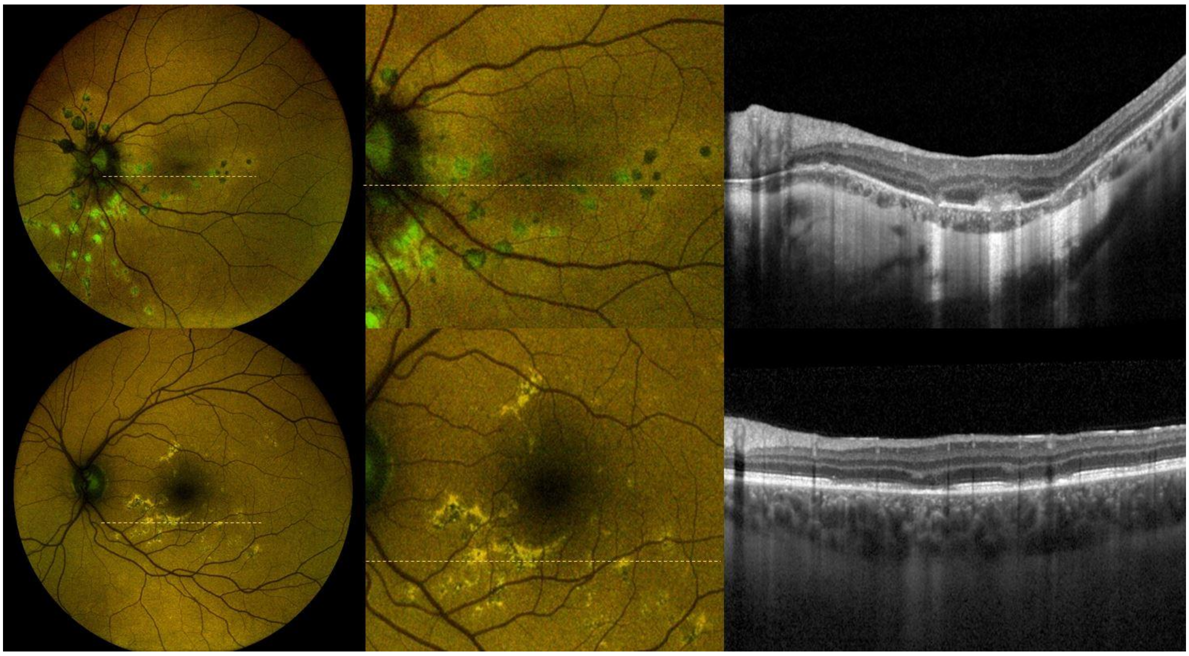

Supplementary Figure S2. Exemplary comparison of OCT and Color-FAF for one eye with punctate inner choroidopathy (PIC) and acute posterior multifocal placoid pigment epitheliopathy (APMPPE)

One exemplary eye with punctate inner choroidopathy (PIC; top row) and acute posterior multifocal placoid pigment epitheliopathy (APMPPE; bottom row) are shown on Color-FAF and on OCT. Lesions secondary to PIC are easily identifiable on Color-FAF and show an interruption of outer retinal layers, including retinal pigment epithelium and Bruch's membrane, on OCT. Lesions due to APMPPE are easily identifiable on Color-FAF as well and show disrupted ellipsoid and interdigitation zone but preserved retinal pigment epithelium and Bruch's membrane.

Supplementary Table S2. Multivariable regression analyses

	Estimate	Std. Error	p
Intercept	8.0×10^{-1}	4.5×10^{-2}	**<.001**
Sarcoidosis	1.2×10^{-1}	5.3×10^{-2}	**.026**
PIC	2.8×10^{-1}	5.5×10^{-2}	**<.001**
APMPPE	9.2×10^{-2}	5.1×10^{-2}	.074
Lens status	-1.0×10^{-3}	5.3×10^{-2}	.334
Inflammatory Activity	1.5×10^{-2}	1.5×10^{-2}	.984

PIC = punctate inner choroidopathy; APMPPE = acute posterior multifocal placoid pigment epitheliopathy

Multivariable regression analyses using linear mixed models including a random intercept for each patient is demonstrated. Estimated variance of random effects was 0.0069. Each subgroup was compared to birdshot chorioretinopathy. Lens status and inflammatory activity were compared for all subgroups.

Supplementary Table S3. Multimodal findings of lesions in posterior uveitis

	Color-FAF (GEFC/REFC Ratio ± SD)	Intensity-normalized Color-FAF	OCT
**Birdshot**	lesions barely visible<br>all lesions: 0.82 ± 0.11<br>active lesions: 0.81 ± 0.04<br>inactive lesions: 0.82 ± 0.10	slightly green	no notable choroidal thinning in fundus areas corresponding to lesions
**Sarcoidosis**	lesions visible<br>all lesions: 0.92 ± 0.11<br>active lesions: 0.92 ± 0.13<br>inactive lesions: 0.92 ± 0.11	moderately green	choroidal thinning in fundus areas corresponding to lesions
**PIC**	lesions visible<br>all lesions: 1.09 ± 0.19<br>active lesions: 1.10 ± 0.18<br>inactive lesions: 1.09 ± 0.15	strongly green	interruption of outer retinal layers, RPE, and Bruch's membrane
**APMPPE**	lesions visible<br>all lesions: 0.87 ± 0.10<br>active lesions: 0.87 ± 0.06<br>inactive lesions: 0.87 ± 0.10	slightly green	disruption of ellipsoid and interdigitation zone with preserved RPE and Bruch's membrane

FAF = fundus autofluorescence; GEFC = green emission fluorescent components; REFC = red emission fluorescent components; SD = standard deviation; OCT = optical coherence tomography; PIC = punctate inner choroidopathy; APMPPE = acute posterior multifocal placoid pigment epitheliopathy

References

1. Wintergerst MWM, Liu X, Terheyden JH, et al. Structural Endpoints and Outcome Measures in Uveitis. *Ophthalmologica*. 2021. doi:10.1159/000517521
2. Bousquet E, Khandelwal N, Séminel M, et al. Choroidal Structural Changes in Patients with Birdshot Chorioretinopathy. *Ocul Immunol Inflamm*. 2021;29(2):346-351. doi:10.1080/09273948.2019.1681472
3. Nussenblatt RB, Whitcup SM, Palestine A. G., eds. *Uveitis: Fundamentals and Clinical Practice*. Mosby; 1996.
4. Sassalos TM, Vitale AT, Conrady CD. Posterior scleritis and acute posterior multifocal placoid pigment epitheliopathy: A case of painful chorioretinitis and review of the current literature. *American Journal of Ophthalmology Case Reports*. 2021;23:101159. doi:10.1016/j.ajoc.2021.101159
5. Steiner S, Goldstein DA. Imaging in the diagnosis and management of APMPPE. *Int Ophthalmol Clin*. 2012;52(4):211-219. doi:10.1097/IIO.0b013e318265d45a
6. Ahnood D, Madhusudhan S, Tsaloumas MD, Waheed NK, Keane PA, Denniston AK. Punctate inner choroidopathy: A review. *Survey of Ophthalmology*. 2017;62(2):113-126. doi:10.1016/j.survophthal.2016.10.003
7. Amer R, Lois N. Punctate inner choroidopathy. *Survey of Ophthalmology*. 2011;56(1):36-53. doi:10.1016/j.survophthal.2010.03.009
8. Ozgonul C, Besirli CG. Recent Developments in the Diagnosis and Treatment of Ocular Toxoplasmosis. *Ophthalmic Res*. 2017;57(1):1-12. doi:10.1159/000449169
9. Saleh M. Placoid pigment epitheliopathy and serpiginous choroiditis. *J Fr Ophtalmol*. 2020;43(2):e55-e66. doi:10.1016/j.jfo.2019.12.001
10. Spaide RF, Goldberg N, Freund KB. Redefining multifocal choroiditis and panuveitis and punctate inner choroidopathy through multimodal imaging. *Retina*. 2013;33(7):1315-1324. doi:10.1097/IAE.0b013e318286cc77
